# Contribution of problem drug users’ deaths to excess mortality in Scotland: secondary analysis of cohort study

**DOI:** 10.1136/bmj.a478

**Published:** 2008-07-31

**Authors:** Michael Bloor, Maria Gannon, Gordon Hay, Graham Jackson, Alastair H Leyland, Neil McKeganey

**Affiliations:** 1 Centre for Drug Misuse Research, University of Glasgow, Glasgow G11 6PW; 2 Vital Events Branch, General Register Office for Scotland, Edinburgh EH12 7TF; 3 MRC Social and Public Health Sciences Unit, Glasgow G12 8RZ

## Abstract

**Objectives** To examine the “Scottish effect”—namely, the growing divergence between mortality in Scotland and England that is not explained by national differences in levels of deprivation—and, more specifically, to examine the extent to which the Scottish effect is explained by cross national differences in the prevalence of problem drug use.

**Design** Secondary analysis of cohort study (the DORIS study).

**Participants** 1033 Scottish drug users recruited to the cohort study in 33 drug treatment facilities across Scotland in 2001-2 and followed up 33 months later in 2004-5.

**Results** 38 deaths occurred in the cohort, giving a standardised mortality ratio for the cohort of 1244 (95% credible interval 876 to 1678). Only 22 of the 38 deaths in drug users were classified as drug related deaths. From estimates of the size of the problem drug using populations in both England and Scotland, the contribution of deaths in drug users to national death rates can be estimated: the attributable risk fraction for Scotland is 17.3% (12.3% to 22.8%) and that for England is 11.1% (7.8% to 14.8%). Excluding estimated numbers of deaths in drug users would bring down age standardised mortality at ages 15-54 years from 196 to 162 per 100 000 in Scotland and from 138 to 122 per 100 000 in England; 32.0% (22.3% to 43.0%) of the excess mortality in Scotland is due to drug use.

**Conclusion** Although problem drug use is a low prevalence risk behaviour, it carries a high mortality; the standardised mortality ratio for Scottish drug users is 12 times as high as for the general population. The higher prevalence of problem drug use in Scotland than in England accounts for a third of Scotland’s excess mortality over England. Successful public health efforts to reduce the prevalence of problem drug use in Scotland or deaths in Scottish drug users would have a dramatic impact on overall mortality in Scotland.

## Introduction

Death rates are known to be higher in Scotland than in England and Wales. In recent years, although Scottish death rates have been falling, the relative difference between the nations has increased: mortality was 12% higher in Scotland than in England and Wales in 1981 but 15% higher in 2001.^1^ In the past, poorer health in Scotland has been attributed to higher levels of deprivation: if local mortality was adjusted for local Carstairs deprivation scores (based on levels of adult male employment, car ownership, social class composition, and overcrowding), 60% of Scotland’s excess mortality in 1981 was explained by greater relative deprivation.^2^ However, by the 1991 census (and continuing at the 2001 census) deprivation was accounting for less than half of Scotland’s excess mortality,^1^ ^3^ and the unaccounted for excess was increasingly marked among the Scottish male population aged 0-44 years.^1^ This growing disproportionality has been dubbed the “Scottish effect.”

Possible explanations for the Scottish effect reflect the wider debates that have taken place on the causes of inequalities in health in the United Kingdom ever since the publication of the Black report in 1980.^4^ Thus, people have suggested that the effect is a measurement artefact, that Carstairs deprivation scores capture relative deprivation less effectively in the 21st century than they did in 1981. Hanlon and colleagues discount the artefact explanation and, pointing to higher levels of alcohol consumption, lower levels of physical activity, and a higher prevalence of smoking in Scotland (compared with England), tentatively suggest that “Scots in an equivalent deprivation category have higher levels of personal risk factors.”^1^ Our purpose here is to endorse that suggestion and posit that a single risk factor—problem drug use—may be responsible for a large part of the observed, deprivation adjusted, cross national differences in rates of premature death.

## Methods

Numbers and trends in “drug related deaths” in Scotland are regularly reported,^5^ but the definition of drug related deaths used in these reports is deliberately and properly a restrictive one, limited to deaths directly due to the pharmacological effect of an illicit drug.^6^ In order to estimate the numbers of deaths in a population of drug users, deaths that embrace not just overdoses from illicit drugs but also deaths from bloodborne infections, from violent assaults, from suicides, and from other events associated with drug use, cohort studies on populations of drug users are needed.^7^ ^8^ DORIS (drug outcomes research in Scotland) is the largest ever repeat interview study of Scottish drug users, involving following up 1033 problem drug users who started a new treatment episode in 33 drug treatment agencies (representing a range of treatment modalities and including treatment in prisons) across Scotland in 2001-2. Of the 1180 problem drug users who were asked to participate, 147 people refused, giving a participation rate of 87.5%; the most common reason given for refusal was lack of time. The DORIS sample comprises one in 11 of the drug users entering treatment in 2001 and returned in the Scottish drug misuse database (a database of all new entrants into drug treatment facilities), and the sociodemographic profile of the sample is comparable to the profile of returns made to that database in 2001.^9^ DORIS respondents had a mean age of 28, and 69% of respondents were male; although misuse of several drugs was the common pattern, the great majority of respondents reported that their main drug was heroin.

We matched data between General Register Office for Scotland (GROS) mortality data and those members of the DORIS sample who were lost to follow-up at 33 months. The Vital Events Branch of GROS then established which of the deaths in the DORIS cohort had previously been classified as a drug related death in earlier annual reports.^5^ We also made inquiries with the Office for National Statistics in England about whether the death of a DORIS sample member who had died in England had been included in their equivalent count of drug related deaths.

Estimates can be made of the total number of deaths in drug users (as opposed to drug related deaths) by combining the standardised mortality ratio from the DORIS cohort with prevalence data on problem drug use (available for both Scotland and England),^10^ ^11^ calculated by using population estimation methods. These methods (identical for both the Scottish and the English prevalence estimates) model the “hidden population” of problem drug users not in contact with services from overlaps between populations of drug users known to a range of different services, including the drug treatment services where the DORIS sample was recruited. Therefore, any possible numerator-denominator bias should be quite limited in extent and estimates of death will include deaths of hidden drug users as well as of those in contact with services. The contribution of deaths among drug users to overall Scottish and English death rates can be calculated as the attributable risk fraction (ARF) in the formula ARF=P_pdu_(SMR/100−1)/(P_pdu_(SMR/100−1)+1), where P_pdu_ is the proportion of the population who are problem drug users and SMR is the standardised mortality ratio for that same subpopulation calculated through indirect standardisation to the Scottish population.^12^

Detailed age and sex breakdowns are not publicly available for the English problem drug user cohort study that is the equivalent of DORIS (the national treatment outcomes research study, NTORS), so we have used the standardised mortality ratios for the DORIS cohort to calculate both the English and the Scottish attributable risk fractions. This seems to be acceptable, as the overall DORIS and NTORS death rates are nearly identical and the NTORS data were gathered at an earlier period (1995-9) than DORIS (2001-4). We calculated standardised mortality rates for England and Scotland by direct standardisation to the European standard population. We estimated the standardised mortality for the non-drug using population in the two countries and, comparing these rates with the observed rates, estimated the proportion of the excess mortality in Scotland that was attributable to drug use. Mortality data and population data came from the Office for National Statistics and the General Register Office (Scotland).^13^ ^14^ ^15^

We report posterior means and 95% credible intervals estimated with WinBUGS,^16^ based on two parallel chains of length 10 000 following a burn-in of 10 000. The credible intervals are ranges of values within which the relevant parameter lies with a probability of 0.95; in this sense, they may be interpreted in a similar manner to confidence intervals. The relevant WinBUGS code can be found on Leyland’s website (www.sphsu.mrc.ac.uk/research_project.php?prjid=BUGSCODE&bcrumbs=MH.METH).

## Results

### Deaths in DORIS cohort

Thirty eight deaths occurred in the 1033 DORIS sample members in the 33 month period, of which only just over half (22) were classified by the General Register Office for Scotland/Office for National Statistics as “drug related deaths.” The cause of death of one cohort member remained unascertained, despite further inquiries by the General Register Office. Of the remaining 15 cohort deaths that were not classed as drug related, six were suicides, including three overdoses (of paracetamol, amitriptyline, and colchicine), one was an overdose of “undetermined intent” involving fluoxetine and propranolol, three were due to an “infection associated with drug abuse” (with a fourth due to endocarditis), two were due to assaults, one was due to “alcoholic liver disease,” and one was due to hypothermia/exposure.

The mortality in the DORIS sample is comparable to rates in other cohort studies of drug users in England (the NTORS cohort) and abroad.^7^ ^8^ Table 1 shows mortality as a crude death rate per 1000 person years, where person years are calculated as the difference between the dates of death and of the initial DORIS interview.

**Table 1 tbl1:** Crude death rates in DORIS sample

	Person years	All deaths	Drug related deaths cohort	Crude death rate (95% CI) per 1000 person years	Crude death rate (95% CI) for drug related deaths cohort
Men	1953	27	15	14 (9 to 20)	7 (4 to 13)
Women	864	11	7	13 (6 to 23)	8 (3 to 17)
Total	2817	38	22	13 (10 to 18)	8 (5 to 12)

### Possible contribution of drug use to Scottish excess mortality

The standardised mortality ratio for men in the DORIS cohort (whose age at recruitment ranged from 16 to 53, with a mean age of 28) was 834 (95% credible interval 549 to 1182) (table 2). In 2002 the age standardised mortality for Scottish men aged 15-54 was 259 per 100 000, and for England it was 173 per 100 000. For the same age group, the prevalence of problem drug use in Scotland in 2003 was 2.69% (95% confidence interval 2.11% to 4.17%).^10^ In England in 2004-5 the prevalence of problem drug use among men aged 15 to 64 was 1.32%.^11^ Assuming that opiate use among men aged 55 to 64 was negligible, this equates to a prevalence of 1.65% among men aged 15 to 54. These data led to an estimated attributable risk fraction of all deaths among male drug users in Scotland of 16.3% (95% credible interval 8.8% to 25.5%) and an attributable risk fraction in England of 10.7% (6.8% to 15.1%). If the estimated deaths in drug users in the two male populations was excluded, the standardised mortality in the two populations would fall from 259 per 100 000 to 217 (95% credible interval 192 to 238) per 100 000 in Scotland and from 173 per 100 000 to 154 (146 to 161) per 100 000 for England. This indicates that 27.5% (95% credible interval 9.3% to 50.3%) of the excess mortality for Scottish men aged 15 to 54 is due to the greater prevalence of problem drug use in Scotland.

**Table 2 tbl2:** Proportion of problem drug users, standardised mortality ratios, attributable risk fractions, age standardised mortality, excess mortality, and proportion of excess due to drug use (with 95% credible intervals unless stated otherwise) in England and Scotland, for men and for men and women combined

	Men			Men and women	
	England	Scotland		England	Scotland
Proportion (%) of problem drug users (95% confidence interval)	1.65*	2.69 (2.11 to 4.17)		1.07 (1.06 to 1.11)	1.84 (1.84 to 2.01)
Standardised mortality ratio in population of drug users (DORIS cohort)	†	834 (549 to 1182)		†	1244 (876 to 1678)
Attributable risk fraction (%)	10.7 (6.8 to 15.1)	16.3 (8.8 to 25.5)		11.1 (7.8 to 14.8)	17.3 (12.3 to 22.8)
Age standardised mortality per 100 000	173 (170 to 175)	259 (250 to 267)		138 (136 to 139)	196 (191 to 201)
Excess mortality per 100 000	–	86 (77 to 95)		–	58 (53 to 64)
Age standardised mortality per 100 000, excluding deaths in drug users	154 (146 to 161)	217 (192 to 238)		122 (117 to 127)	162 (150 to 173)
Excess mortality per 100 000, excluding deaths in drug users	–	63 (42 to 80)		–	40 (32 to 47)
Proportion of excess mortality due to drug use (%)	–	27.5 (9.3 to 50.3)		–	32.0 (22.3 to 43.0)

*95% confidence interval not given.

†Assumed to be same as for DORIS cohort.

The overall (in men and women) standardised mortality ratio for the DORIS cohort is 1244 (95% credible interval 876 to 1678), and the overall attributable risk fraction is 17.3% (12.3% to 22.8%) for Scotland and 11.1% (7.8% to 14.8%) for England. The corresponding prevalences of problem drug use are 1.84% (95% confidence interval 1.84% to 2.01%) for Scotland and 1.07% (1.06% to 1.11%) for England. Exclusion of the estimated deaths in drug users resulted in falls in the standardised mortality from 196 per 100 000 to 162 (95% credible interval 150 to 173) per 100 000 in Scotland and from 138 per 100 000 to 122 (117 to 127) per 100 000 in England. This suggests that 32.0% (95% credible interval 22.3% to 43.0%) of the excess Scottish mortality is due to the greater prevalence of problem drug use in Scotland.

## Discussion

Our data suggest that one particular risk behaviour, problem drug use, accounts for a third of excess mortality in Scotland compared with England among people aged 15 to 54, supporting Hanlon and colleagues’ suggestion that the “Scottish effect” can be explained by higher prevalences of risk behaviours in Scotland than in England within a particular level of deprivation. Note also that a rapid increase in problem drug use (and particularly heroin use) occurred in the 1980s in Scotland,^17^ at the very point at which deprivation measures (Carstairs deprivation scores) began to account for less than half of the cross national variance in rates.

### Uncertainties and limitations

Uncertainty intervals are attached to these various estimates. For Scottish men aged 15-54 (for whom the “Scottish effect” is strongest), deaths in drug users could be accounting for as much as half or as little as a tenth of the excess Scottish mortality; however, intervals are narrower when deaths in men and women are combined. The time periods of the different datasets used are not wholly matched; although we have been able to use datasets from contiguous time periods, readers must decide on the windows of applicability of the different datasets.

The definitions of drug users used in the DORIS study and the two different prevalence estimates differ slightly: eligibility in the DORIS study was simply a matter of starting a new episode of drug treatment; in the English prevalence study the estimate related to use of opiates, crack/cocaine, or both; in the Scottish prevalence study the estimate related to use of opiates, benzodiazepines, or both. Such differences in definition raise the possibility of numerator-denominator biases in mortality calculations, but the practical importance of definitional differences in this instance is small—for example, although the DORIS sample might have started treatment because of misuse of a range different substances other than opiates or benzodiazepines, in fact 88% of the sample had used heroin in the three months before recruitment.

The formula used to calculate the attributable risk fraction has been judged to be suitable when no confounding of the exposure-disease association exists,^18^ but we cannot explore the possibility of such confounding in these data. The mortality in DORIS is consistent with those found in studies elsewhere,^7^ ^12^ and the DORIS sample is the largest repeat interview cohort study of Scottish drug users. Thus, although the sample represents less than a tenth of the annual total of people seeking treatment for drug use in Scotland, our claim that mortality in the DORIS sample indicates that deaths in drug users represent an important fraction of deaths in the Scottish 15-54 year old population is warranted. However, a larger follow-up study of drug users would provide more certain estimates of mortality in drug users.

### Implications

Problem drug use is a low prevalence risk behaviour compared with tobacco consumption, high alcohol consumption, and low levels of physical activity, but it carries a high mortality: mortality in the DORIS sample was 12 times that of the general Scottish population aged 15 to 54. This high mortality is not fully reflected in published reports on “drug related deaths,” as definition of such deaths is deliberately and properly a restrictive one, limited to overdoses from illicit drugs and not the wider spectrum of deaths (including deaths from infections, assaults, and overdose from drugs such as paracetamol) found in the DORIS sample. In the DORIS sample, only just over half the deaths that occurred among drug users were classed as drug related deaths. It follows that deaths in problem drug users are a potentially important contributor to national and local mortality.^19^ ^20^

Explorations of health inequalities, reflected in differential mortality, thus need to embrace studies of differences in the prevalence of problem drug use. The identification of an important role for problem drug use in understanding excess mortality does not, of course, decrease the importance of the link between socioeconomic deprivation and poor health, as the association between relative deprivation and uptake of drugs is long established.^6^ ^21^ ^22^

Successful public health initiatives to reduce the prevalence of problem drug use or to reduce deaths among drug users would have a strong impact on overall mortality in both Scotland and England. Policy efforts to reduce health inequalities could and should make drug misuse a prime target, and the need to tackle drug misuse as part of a strategy to reduce health inequalities was recognised in the report of the Ministerial Task Force on Health Inequalities published on 19 June this year^23^. The evidence is mixed on how successful current public health initiatives have been in Scotland. No trend data on deaths in Scottish drug users are available, but that fraction of such deaths that are classed as drug related deaths has continued to rise.^24^ However, the latest Scottish prevalence estimates show a small fall in the prevalence of problem drug use between 2000 and 2003.^10^

**What is already known on this topic**

The excess mortality in Scotland over that in England is greater than can be accounted for by higher deprivation rates
Mortality among drug users is greatly in excess of that in comparable age matched populations

**What this study adds**

Only just over half the deaths in the largest ever repeat interview study of Scottish drug users were officially reported as drug related deaths
A third of the “excess” mortality in Scotland can be accounted for by Scotland’s higher prevalence of problem drug use

## Notes

We thank the interviewers and the study participants; Carole Bain and Tommy O’Brien for their contribution to the fieldwork; Colin Carroll for data processing; and the DORIS study advisory group members for their advice.

Ethical approval: Ethical oversight of the DORIS study was exercised by the Scottish Multi-Centre Research Ethics Committee, which also granted permission to apply to the General Register Office for Scotland (GROS) to establish which deaths had occurred among members of the cohort who had been lost to follow-up at 33 months. The GROS application was approved by the medical adviser to the registrar general and by the Privacy Advisory Committee.

Provenance and peer review: Not commissioned; externally peer reviewed.

## Notes

**Cite this as:** *BMJ* 2008;337:a478
